# Neutrophil Targeting Platform Reduces Neutrophil Extracellular Traps for Improved Traumatic Brain Injury and Stroke Theranostics

**DOI:** 10.1002/advs.202308719

**Published:** 2024-03-23

**Authors:** Qingchun Mu, Kai Yao, Madiha Zahra Syeda, Jinlong Wan, Qian Cheng, Zhen You, Rui Sun, Yufei Zhang, Huamiao Zhang, Yuting Lu, Zhicheng Luo, Yang Li, Fuyao Liu, Huiping Liu, Xinyu Zou, Yanfen Zhu, Kesong Peng, Chunming Huang, Xiaoyuan Chen, Longguang Tang

**Affiliations:** ^1^ Gaozhou People's Hospital Maoming 525200 China; ^2^ Department of Neurosurgery First Affiliated Hospital of Harbin Medical University Harbin 150001 China; ^3^ St. Michael's Hospital Fully Affiliated Hospital of University of Toronto Toronto Ontario M5B 1W8 Canada; ^4^ Basic Medical College Guilin Medical University Guilin 541199 China; ^5^ Liangzhu Laboratory Zhejiang University 1369 West Wenyi Road Hangzhou 311121 China; ^6^ School of Pharmaceutical Sciences Guangdong Provincial Key Laboratory of New Drug Screening Southern Medical University Guangzhou 510515 China; ^7^ Department of Pharmacy, Center for Regeneration and Aging Medicine the Fourth Affiliated Hospital of School of Medicine, and International School of Medicine, International Institutes of Medicine Zhejiang University Yiwu 322000 China; ^8^ Key Laboratory for Advanced Drug Delivery Systems of Zhejiang Province College of Pharmaceutical Sciences Zhejiang University Hangzhou 310058 China; ^9^ Departments of Diagnostic Radiology Chemical and Biomolecular Engineering and Biomedical Engineering Yong Loo Lin School of Medicine and College of Design and Engineering National University of Singapore Singapore 119074 Singapore; ^10^ Clinical Imaging Research Centre Centre for Translational Medicine Yong Loo Lin School of Medicine National University of Singapore Singapore 117599 Singapore; ^11^ Nanomedicine Translational Research Program Yong Loo Lin School of Medicine National University of Singapore Singapore 117597 Singapore

**Keywords:** neutrophil, neutrophil extracellular traps (NETs), stroke, theranostics, traumatic brain injury

## Abstract

Traumatic brain injuries (TBI) and stroke are major causes of morbidity and mortality in both developing and developed countries. The complex and heterogeneous pathophysiology of TBI and cerebral ischemia‐reperfusion injury (CIRI), in addition to the blood‐brain barrier (BBB) resistance, is a major barrier to the advancement of diagnostics and therapeutics. Clinical data showed that the severity of TBI and stroke is positively correlated with the number of neutrophils in peripheral blood and brain injury sites. Furthermore, neutrophil extracellular traps (NETs) released by neutrophils correlate with worse TBI and stroke outcomes by impairing revascularization and vascular remodeling. Therefore, targeting neutrophils to deliver NETs inhibitors to brain injury sites and reduce the formation of NETs can be an optimal strategy for TBI and stroke therapy. Herein, the study designs and synthesizes a reactive oxygen species (ROS)‐responsive neutrophil‐targeting delivery system loaded with peptidyl arginine deiminase 4 (PAD4) inhibitor, GSK484, to prevent the formation of NETs in brain injury sites, which significantly inhibited neuroinflammation and improved neurological deficits, and improved the survival rate of TBI and CIRI. This strategy may provide a groundwork for the development of targeted theranostics of TBI and stroke.

## Graphical abstract

Neutrophils and neutrophil extracellular traps (NETs) play a very important role in TBI and stroke. A neutrophil‐hitchhiking delivery system loaded with PAD4 inhibitor is developed to prevent the formation of NETs in brain injury sites, which significantly inhibits the neuroinflammation, improves the neurological deficits, and prolongs the survival of TBI and stroke.

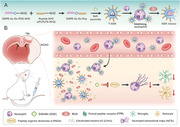

## 1 Introduction

Traumatic brain injury (TBI) and Stroke are among the leading causes of disability in the world, but treatment options are limited.^[^ ^1^, ^2^ ^]^ Over sixty million cases of TBI are reported each year, and the incidence has increased over time.^[3^ ^]^ TBI has complex pathological characteristics that can lead to a variety of psychiatric disorders in the future.^[4^ ^]^ Approximately 85% of all strokes are ischemic strokes, which are caused by a thrombus that obstructs a blood vessel.^[5^ ^]^ Current FDA/EMA‐approved therapies for TBI and acute ischemic stroke are constrained by a narrow therapeutic window, selective efficacy, hemorrhagic complications, and other disadvantages.^[^ ^6^ ^]^

Secondary injuries are a delayed response to a primary TBI. It includes inflammation, oxidative stress, mitochondrial dysfunction, and cell death.^[1^ ^]^ The migration and organized infiltration of immune cells into the brain parenchyma is promoted by a sterile inflammatory response following TBI or ischemic damage.^[^ ^7^, ^8^, ^9^ ^]^ Recent studies have also shown that infiltrating neutrophils, at the site of injury, release nuclear DNA and chromatin strands studded with granular proteins to make cloud‐like structures called neutrophil extracellular traps (NETs).^[10^ ^]^ The process is known as NETosis. Peptidyl arginine deiminase 4 (PAD4), a member of the PAD enzyme family, plays a crucial role in NETosis by citrullinating histone‐3 (CitH3), which results in the condensation and release of neutrophil chromatin.^[^ ^11^, ^12^, ^13^ ^]^ Recent reports have indicated that PAD4 activation is increased in the ischemic disorders,^[^ ^14^, ^15^ ^]^ and that inhibition of PAD4 prevents the ischemic injury in certain organs. Therefore, genetic or pharmacological inhibition of NETosis is effective in preventing ischemic damage and brain injury.

Recent studies have shown that PAD4 inhibitors can be used to treat ischemic disorders. However, the short half‐life of therapeutic reagents, poor solubility, and low bioavailability, as well as metabolic toxicity, limit their efficacies. Consequently, it is essential to develop new and enhanced strategies for drug delivery to ischemic regions utilizing modern pharmaceutics. Due to their prolonged bioavailability, increased solubility, and neuroprotective effects via the blood‐brain barrier (BBB) by reducing inflammatory responses, nanoparticles can effectively address these issues.^[16^ ^]^ To improve the targeting of inflammatory sites, neutrophils and neutrophil‐derived cell membranes have been utilized as drug delivery vehicles.^[^ ^16^, ^17^ ^]^ Large‐scale production of neutrophil cell membranes is, however, inconvenient and expensive.

**Figure 1:**
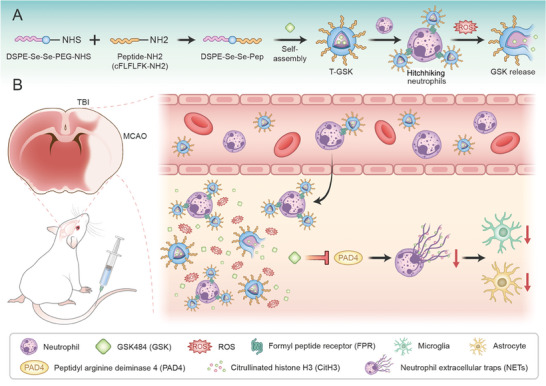
Neutrophil‐targeting drug‐delivery system actively delivers drugs into the brain injury sites. Schematic diagram of A) synthesis of neutrophil‐targeting, ROS responsive therapeutic GSK (T‐GSK), and B) their mechanistic targets in injured brain tissues.

Herein, we designed a nanocarrier by conjugating a neutrophil selective binding peptide cinnamyl‐F‐(D)L‐F‐(D)L‐F (CFLFLF) with reactive oxygen species (ROS)‐responsive polymer DSPE‐Se‐Se‐PEG.^[18^ ^]^ The nanoparticles contained GSK484, an inhibitor with a strong preference for PAD4 over other members of the PAD enzyme family.^[19^ ^]^ In addition, the shell of the nanoparticles contained a polymer that can break bonds when exposed to ROS (**Figure** **1**A). The ability of the designed nanoparticles to bind to the surface of neutrophils in the bloodstream and at the site of injury was assessed. In addition, the nanoparticles' efficacy, targeted delivery, and GSK484 release were evaluated. In mouse models of TBI and middle cerebral artery occlusion (MCAO), this strategy demonstrated remarkable neuroprotective effect in vivo (Figure 1B).

## 2 Results and Discussion

### 2.1 Neutrophil Count Correlates with Deteriorating Neurological Function in Patients with Severe Brain Injuries

**Figure 2:**
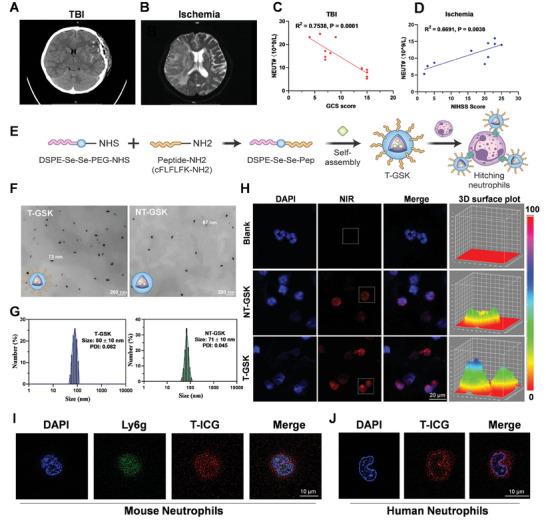
Elevated neutrophil counts in patients with severe brain injuries and the preparation of T‐GSK nanoparticles that can specifically bind to neutrophils in vitro. A) Axial computed tomography (CT) scans show left frontal and temporal lobe contusion from a representative patient (6‐year‐old male, GCS = 7), brought to the hospital 7 h after car accident. B) The axial apparent diffusion coefficient (ADC) map shows dark signal in right cerebral hemisphere of a representative patient (70‐year‐old woman, NIHss = 20) who was admitted to hospital 6 h after being found unconscious. C) Correlation analysis between patient neutrophil count and Glasgow Coma Scale (GCS) score, or D) NIH stroke scale (NIHss) score. Pearson's coefficient (R) and P value are shown as insets. E) Schematic diagram of the preparation of T‐GSK and hitchhiking neutrophils through formyl peptide receptor (FPR). F,G) Representative TEM (F) and DLS images (G) of T‐GSK and NT‐GSK nanoparticles. H) Confocal images of activated neutrophils for the uptake of ICG‐labeled T‐GSK and NT‐GSK in vitro. I) Representative confocal images showing the binding of nanoparticles (T‐ICG) on the surface of mouse neutrophils (Ly^6^G). J) Representative confocal images showing the binding of nanoparticles (T‐ICG) on the surface of human neutrophils.

In the clinic, a correlation existed between neutrophil count and brain injuries (**Figure** **2**A–D). There was a statistically significant inverse correlation between the Glasgow coma scale (GCS), a clinically valid measure of neurological function, and the neutrophil count of TBI patients (R^2^ = 0.7538; P = 0.0001) (Figure 2C). Similarly, the NIH stroke scale/score (NIHss), a measure of stroke severity, was significantly correlated with the neutrophil count in stroke patients (Figure 2D, R^2^ = 0.6691, P = 0.001). The significance of neutrophils in determining the severity of brain injury is suggested by these findings.

### 2.2 Rational Design and In Situ Synthesis of Nanomedicine Targeting Neutrophils

We designed and synthesized a ROS‐responsive polymer (DSPE‐Se‐Se‐PEG) by introducing the ROS‐cleavable ‐Se‐Se‐ group as an intermediate chain between 1, 2‐distearoyl‐sn‐glycero‐3‐phosphoethanolamine (DSPE) and poly(ethylene glycol) (PEG). DSPE‐PEG is a biocompatible, biodegradable, amphiphilic material that can be functionalized with a variety of biomolecules for desired functions. The carboxyl terminus of PEG had been combined with a polypeptide CFLFLFK‐NH2 that targets the formylpeptide receptor (FPR) on neutrophils. The peptide‐modified polymer DSPE‐Se‐Se‐Pep was used to encapsulate the therapeutic drug GSK484 (GSK), which can self‐assemble into nanoparticles (T‐GSK, Figure 1A). After intravenous administration, the nanomedicine (T‐GSK) was rapidly transported by neutrophils to the brain injury site, where the levels of inflammatory ROS were elevated. ROS severed the intermediate ‐Se‐Se‐ bond and destroyed the nanoparticles shell, causing the rapid release of the encapsulated GSK. The ROS, level of microglia, astrocyte‐induced neuroinflammation, and other adverse reactions of brain injury returned to normal (Figure 1B) when drugs were released in real‐time and in a targeted manner at the site of brain injury.

### 2.3 Characterization of T‐GSK Nanoparticles

Figure 2E depicts a schematic illustration of the nanocarrier synthesis. For comparison, we prepared non‐target GSK (NT‐GSK) by self‐assembly of DSPE‐Se‐Se‐mPEG and GSK, similar to T‐GSK. NT‐GSK is incapable of binding to neutrophils because it lacks the specific neutrophil binding peptide. Using transmission electron microscopy (TEM), the average sizes of T‐GSK and NT‐GSK nanoparticles were determined to be 73 and 67 nm, respectively (Figure 2F). Dynamic light scattering (DLS) was used to detect the morphology of nanoparticles, which revealed spherical particles with uniform distribution (Figure 2G). The zeta potentials of T‐GSK and NT‐GSK were also determined to be negative (Figure S1A, Supporting Information). Nanoparticles of T‐GSK were incubated with or without H_2_O_2_ to determine their ROS‐responsiveness and drug‐release behavior. The results demonstrated that the rate of GSK release in the presence of H_2_O_2_ was significantly higher and more rapid than that in the absence of H_2_O_2_ (Figure S1B, Supporting Information). The morphological stability of T‐GSK nanoparticles in aqueous solution at temperatures below 4 °C was maintained over a 7‐day storage period (Figure S1C, Supporting Information).

### 2.4 Measurement of Nanoparticles Hitchhiking on Neutrophils

To assess the uptake of nanoparticles by neutrophils, we incubated activated neutrophils with indocyanine green (ICG)‐labeled nanoparticles and observed them under confocal microscope. Compared to those in the NT‐GSK and control groups, neutrophils in the T‐GSK nanoparticles group exhibited bright red fluorescence. This suggests a larger uptake of T‐GSK nanoparticles by neutrophils (Figure 2H). The binding of nanoparticles on neutrophils' surfaces was also confirmed in the confocal microscopy images (Figure 2I). The internalization of ICG‐labeled nanoparticles by neutrophils was evaluated using flow cytometry, and the results were consistent with the confocal microscopy findings (Figure S2, Supporting Information). To further explore if the designed nanoparticles could bind to human neutrophils, the neutrophils were isolated from human blood and incubated with the nanoparticles. Figure 2J demonstrated that nanoparticles bound significantly to the surface of neutrophils. In addition, flow cytometric results confirmed that nanoparticles bound to human neutrophils (Figure S3, Supporting Information). The dichlorodihydrofluorescein diacetate (DCFH‐DA) assay was then used to demonstrate the efficiency of T‐GSK in ROS clearance in cells following H_2_O_2_ stimulation (Figure S4, Supporting Information).

### 2.5 In Vivo Penetration and Accumulation of ICG‐Labeled Nanoparticles at the Site of Injury

**Figure 3:**
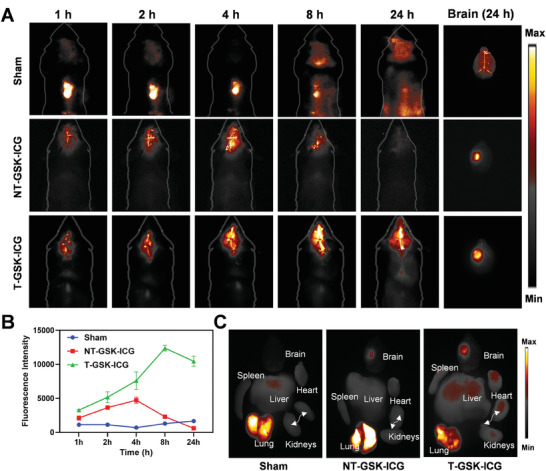
Near‐infrared II region (NIR‐II) imaging results of T‐GSK‐ICG and NT‐GSK‐ICG in TBI model, and of sham. A) Representative NIR‐II images of mice in the T‐GSK‐ICG, NT‐GSK‐ICG, and sham groups (T‐GSK‐ICG) were recorded at the indicated time points after *i.v*. injection. The excised brain NIR‐II imaging was performed at 24 h. Results showed a significant accumulation of T‐GSK‐ICG in the brain of TBI model. B) The quantitative analysis of NIR‐II signals intensity of the brain issue in each group at indicated time points (n = 3/group). Data are represented as mean ± SD C) Ex vivo NIR‐II imaging of major organs (brain, spleen, heart, liver, lungs, and kidneys) excised from mice in different treatment groups.

Recently, near‐infrared II (NIR‐II, 1000–1700 nm) biomedical fluorescence imaging has been developed for non‐invasive in vivo biological imaging that enables deeper and more precise detection of biological tissues or organs than NIR I imaging.^[^ ^20^, ^21^ ^]^ The precise delivery and targeting efficiency of T‐GSK and NT‐GSK to injured brain tissues in vivo was evaluated using NIR‐II imaging. To this end, nanoparticles were synthesized by encapsulating the FDA‐approved near‐infrared fluorochrome indocyanine green (ICG) and injected intravenously to reach the site of inflammation in the injured brain region of mice from different groups at the same dose. At 1, 2, 4, 8, and 24 h after injection, noninvasive NIR‐II imaging of the injured brains was performed. Dynamic NIR images presented the highest fluorescence intensity in the T‐GSK treated group, indicating the highest accumulation of nanoparticles in the T‐GSK group compared to NT‐GSK and sham‐treated groups. This implies the delivery and precise targeting of T‐GSK to damaged brain tissue. Finally, the entire brain from the euthanized mice was removed to perform ex vivo imaging and observe the fluorochrome retention in designated groups (**Figure** **3**A). The quantitative data also revealed that T‐GSK‐treated mice displayed the highest fluorescence intensity, which increased over time and peaked 8 h after injection (Figure 3B). The imaging of additional vital organs ex vivo was then performed. Figure 3C demonstrated that the results were consistent with those of in vivo imaging, confirming that T‐GSK was effective at targeting injury sites. The accumulation of nanoparticles at the site of injury demonstrated that T‐GSK was more likely to cross the damaged BBB and enter the injured brain site than NT‐GSK.

### 2.6 Neuroprotective Effects of T‐GSK against Traumatic Brain Injury In Vivo

**Figure 4:**
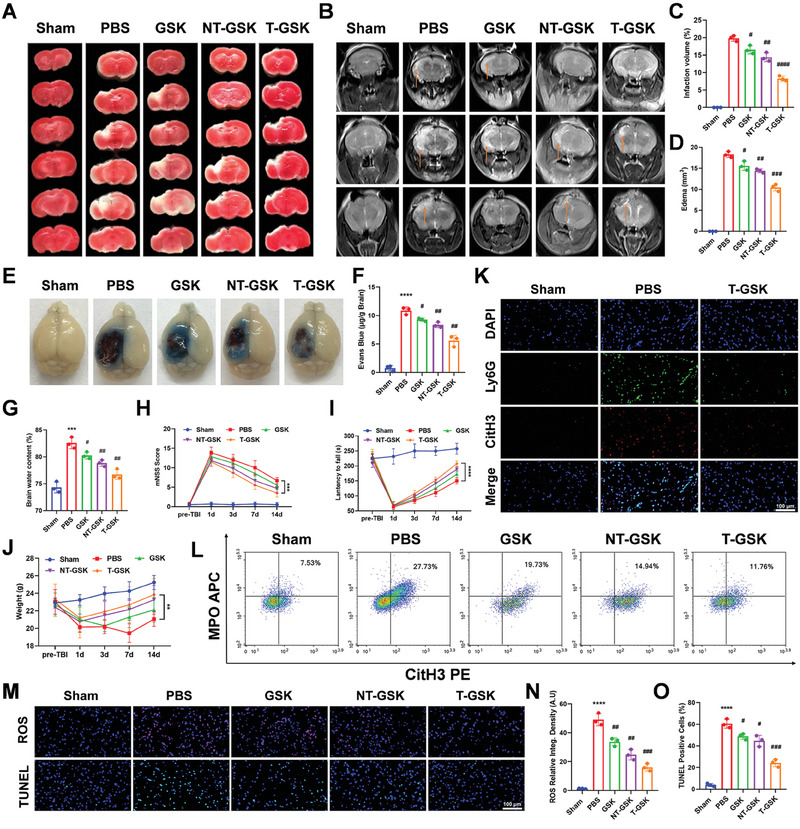
T‐GSK reduces brain tissue infarction, brain edema, penetrability of Evans Blue (EB), and neurological deficits, via inhibiting NETs formation and ROS expression in TBI model. A) T‐GSK significantly reduced the brain infarction evaluated by TTC staining on day 3 after the TBI in mice. B) Brain edema was evaluated by magnetic resonance *T*_2_‐weighted imaging which showed that T‐GSK significantly reduced the size of high‐signal area in the brain tissue on *T*_2_ image. C,D) Quantitative analysis of cerebral infarction percentage calculated from TTC (C), and brain edema calculated from MRI (D). E) EB staining was used to detect the degree of damage to the blood‐brain barrier (BBB). T‐GSK notably reduced the extravasation of EB in brain tissue. F) Quantitative analysis of the concentration of EB in the brain tissue. G) The amount of brain edema was calculated from the ratio of dry to wet weights at 72 h after TBI, which showed that T‐GSK remarkably reduced the water content of brain tissue. (H, I) After mouse TBI, the neurological recovery of mice in each group at different time points was evaluated by mNSS (H) and rotarod test (I). J) Monitoring the effects of GSK, NT‐GSK, and T‐GSK on the body weight of mice at different time points. K) Immunofluorescence staining of the brain tissue sections stained with neutrophils marker (Ly6G) and NETs marker (CitH3) showing the NETs formation in different groups. L) Flow cytometric analysis of MPO release and NETs formation in neutrophils in the indicated groups. M) Immunofluorescence staining of the brain tissue sections shows the ROS expression and TUNEL positive cells in different experimental groups. N,O) Quantitative data shows the decrease of ROS and the higher anti‐apoptotic potential of T‐GSK over NT‐GSK. The data represent the mean ± SD, analyzed by one‐way analysis of variance (ANOVA) with post‐hoc analysis and two‐sided Student's t‐test; *n* = 3–6 biologically independent samples; ^***^ *p* < 0.001 versus Sham; ^****^ *p* < 0.0001 versus Sham; ^#^ *p* < 0.05 versus PBS. ^##^ *p* < 0.01 versus PBS, ^###^ *p* < 0.001 versus PBS, ^####^ *p* < 0.0001 versus PBS.

Inspired by the above findings, we investigated the neuroprotective potential of T‐GSK against TBI‐induced brain damage in a mouse model. We established the mouse model of TBI, and a series of subsequent experiments were conducted to evaluate the cerebral protection offered by respective treatments in each group. Images of brain sections stained with 2,3,5‐triphenyltetrazolium (TTC) revealed extensive damage in the mouse TBI model (PBS‐treated) compared to the sham group. The size of the infarction was significantly reduced in the treatment groups, with the T‐GSK group exhibiting the least damage to brain tissue (**Figure** **4**A). Quantitative analysis of infarct size volume (Figure 4C) revealed the highest percentage in the PBS group and a significant reduction in the T‐GSK group. Several hours after TBI, vasogenic brain edema and BBB damage were observed. This might lead to increased intracranial pressure and decreased cerebral blood perfusion, resulting in secondary cerebral ischemia and worsening the primary injury, clinical decline, and neurological dysfunction.^[^ ^22^, ^23^, ^24^ ^]^ We utilized *T*_2_‐weighted magnetic resonance imaging (MRI) to assess the preventative efficacy of various treatments for cerebral edema. The degree of edema was determined using a *T*_2_‐weighted imaging scan to observe the secondary craniocerebral injury on day 3 post‐TBI. The MRI images of mice treated with GSK, NT‐GSK, and T‐GSK showed a significant decrease in brain edema. T‐GSK treatment resulted in the lowest volume of brain edema compared to the other treatments (Figure 4B), indicating that T‐GSK has a high potential to prevent brain edema. The quantitative analysis of the MRI images in Figure 4D also confirmed the effectiveness of T‐GSK in preventing the development of edema. Next, we evaluated the capacity of T‐GSK to reverse BBB damage caused by TBI. To achieve this, we measured the permeability of Evans blue (EB) in the brains of several mouse strains. On day three following TBI, the PBS group demonstrated a significant increase in EB permeability compared to the control group (Figure 4E). Treatment with GSK, NT‐GSK, and T‐GSK significantly decreased the permeability of EB in TBI brains. Figure 4F demonstrated that the quantitative data of EB staining and the visual observations of EB permeability were in accordance to each other. T‐GSK was more effective than GSK and NT‐GSK in protecting the BBB from trauma‐induced damage. The water content of the injured ipsilateral brain tissue in the PBS group was significantly greater than that in the sham group, whereas the GSK, NT‐GSK, and T‐GSK groups demonstrated a significant decrease in the water content of the injured brain tissue (Figure 4G).

In addition, the modified neurological severity score (mNSS) and the rotating rod experiment were utilized to assess the effectiveness of T‐GSK in preventing motor dysfunction and promoting recovery (Figure 4H). PBS‐treated mice exhibited a higher mNSS score and neurological dysfunction, which gradually improved with T‐GSK treatment, resulting in a lower mNSS score. The T‐GSK group was able to remain on the rotating road significantly longer than the PBS group following TBI, as shown in Figure 4I. These results demonstrated the efficacy of T‐GSK in treating TBI‐related injuries, including cerebral infarction, cerebral edema, and BBB damage. In addition, our findings indicated that T‐GSK could repair neurological deficits and motor dysfunction resulting from traumatic brain injury. To assess the efficacy of T‐GSK, the weight changes resulting from TBI were also recorded. On days 1 and 3 post‐TBI, the weight of mice that had undergone surgery or sustained a brain injury decreased substantially. Mice in the PBS group continued to lose weight until day 7 following injury, while mice in the other treatment groups gained weight gradually until the end of the experiment on day 14 (Figure 4J). This data demonstrated further the observable benefits of T‐GSK in accelerating recovery from TBI. Due to the importance of drug safety in the development of new therapeutics, we performed hematoxylin and eosin (HE) staining on tissue sections of each experimental group's vital organs to observe any pathological changes following drug administration (Figure S5, Supporting Information). Images stained with HE did not reveal any pathological changes, indicating that T‐GSK did not induce any observable side effects or toxicity in the normal organs of mice. In addition, we performed the blood hemolysis experiment to confirm the effect of nanoparticles on hemolysis of blood cells (Figure S6, Supporting Information). The results demonstrated that nanoparticles are typically safe for blood cells.

### 2.7 Efficiency of T‐GSK against NET Formation

Neutrophils are the initial cells to arrive at an injury site. At the site of injury, neutrophils release myeloperoxidase (MPO) and tumor necrosis factor (TNF), destroying the blood‐brain barrier (BBB).^[^ ^25^, ^26^ ^]^ It has also been shown that the formation of NETs by neutrophils exacerbates acute neurological injury following TBI.^[27^ ^]^ Since it was recently reported that GSK484 inhibits PAD4 (a key player in NETosis), we evaluated T‐GSK's ability to inhibit the formation of NETs following TBI. Flow cytometry was utilized to measure the MPO and CitH3 (a marker of NETosis) expression in blood neutrophils. These findings suggest the formation of NETs. Nevertheless, treatment with GSK, NT‐GSK, and T‐GSK reduced the incidence of NETosis, with T‐GSK being the most effective (Figure 4L). Neutrophils (Ly6G marker) were co‐stained with the NETosis marker CitH3 via immunofluorescence, providing additional validation. Figure 4K’s immunofluorescence results demonstrated a significant decrease in CitH3 upon treatment with T‐GSK compared to the PBS‐treated group, confirming the flow cytometry findings. Overall, these results not only confirmed the efficacy of PAD4 inhibition by GSK in preventing NETosis, but also supported the greater potential of T‐GSK in preventing TBI‐induced NET formation.

At the injury site, elevated ROS levels have been linked to TBI. Neutrophils that arrive at a wound site frequently exacerbate the condition by releasing MPO and elevating ROS levels. Our research uncovered consistent evidence of a significant rise in ROS levels after TBI. ROS levels were decreased by the treatments indicated. Compared to other treatments, T‐GSK significantly decreased ROS levels (Figure 4M–N). These results prompted us to investigate the effects of T‐GSK on TBI‐induced brain cell damage and death. The TdT‐mediated dUTP nick‐end labeling (TUNEL) assay was utilized to examine brain tissue sections. As measured by TUNEL, the PBS group had a greater number of apoptotic cells than the control group. Treatment with GSK, NT‐GSK, and T‐GSK significantly reduced the number of apoptotic cells in these groups, with the greatest reduction occurring in the T‐GSK group (Figure 4M,O). These results demonstrate that an increase in ROS following TBI cleaves ROS‐sensitive bonds in T‐GSK and releases GSK at the injury site, which can then inhibit apoptosis and provide neuroprotection.

### 2.8 Inhibitory Effects of T‐GSK on TBI Induced Neuroinflammation and Immune Response

Astrocytes and microglia play essential roles in the brain's response to trauma.^[^ ^28^, ^29^ ^]^ The proliferation of microglia and astrocytes results in an abundance of proinflammatory mediators and neurotoxic molecules, which contribute to the inflammatory response and exacerbate the injury.^[30^ ^]^ Microglia that have been activated can produce pro‐inflammatory cytokines such as interleukin‐6 (IL‐6) and tumor necrosis factor (TNF). These factors promote the activation and proliferation of microglia and astrocytes, thereby amplifying the inflammatory response and creating a positive feedback loop.^[^ ^28^, ^29^ ^]^ Immunofluorescence staining of mouse brain tissue revealed that traumatic brain injury increased the levels of neutrophils, microglia, and astrocytes in comparison to the control group (Figure S7A, Supporting Information). The T‐GSK treatment reduced microglia, astrocytes, and neutrophils significantly (Figure S7B, Supporting Information). In addition, we used enzyme‐linked immunosorbent assay (ELISA) to evaluate the expression of inflammatory cytokines after TBI induction and subsequent treatments. The results demonstrate a significant elevation in the expression of IL‐6 and TNF‐α after TBI compared to the sham group, which was significantly reduced after treatment with GSK, NT‐GSK, and T‐GSK (Figure S7C, Supporting Information). These findings suggest that nanoparticles can effectively target the injury site and modulate post‐injury inflammatory responses by lowering pro‐inflammatory cytokine levels.

### 2.9 Evaluation of the Efficacy of T‐GSK against Cerebral Ischemia (MCAO)

**Figure 5:**
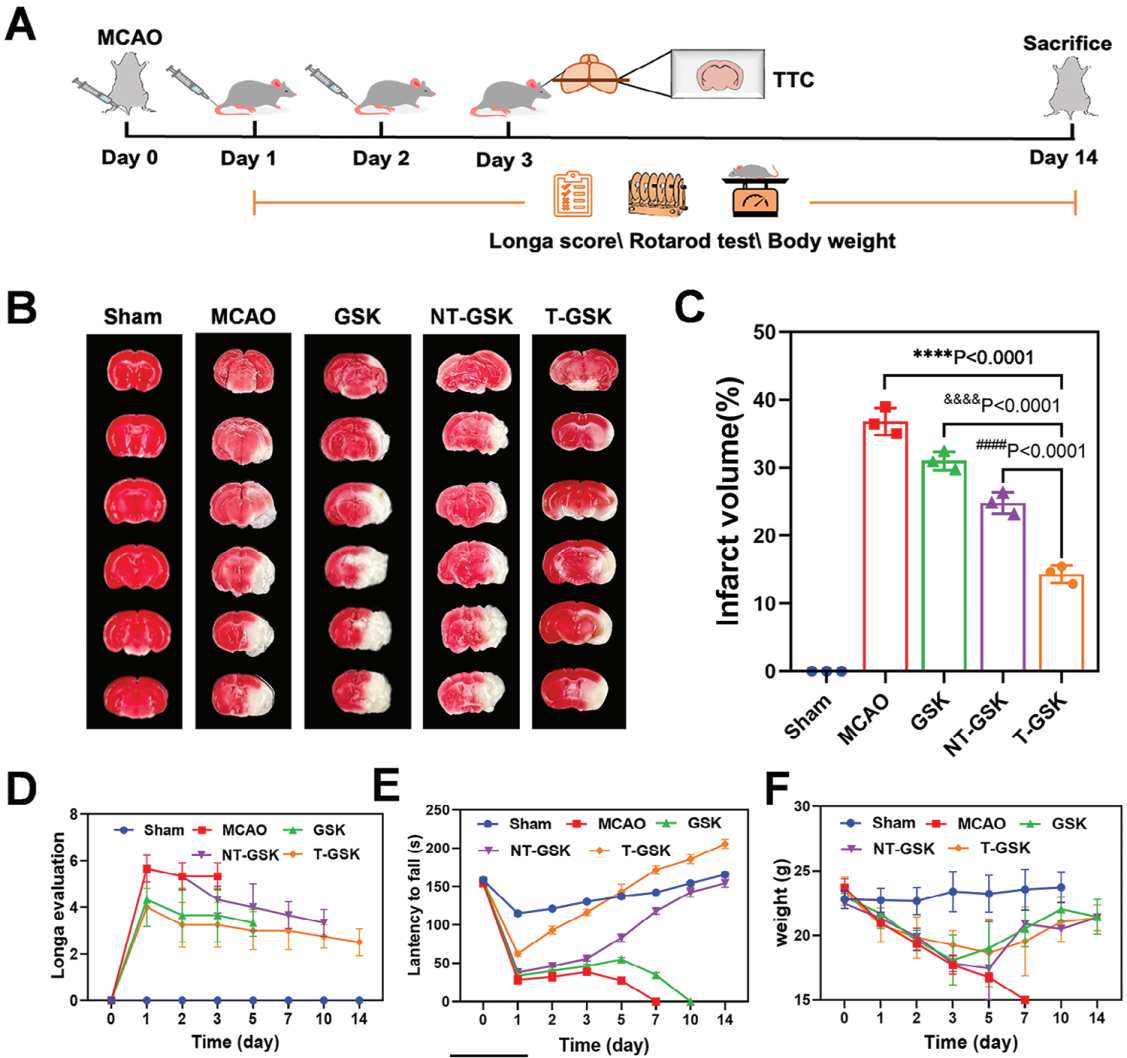
T‐GSK reduces brain tissue infarction and neurological deficits induced by MCAO. A) Protocol for drug administration. Tail vein injections of vehicle or GSK (8 mgkg^−1^) and nanoparticles were performed at MCAO onset, 24 h, and 48 h after occlusion, respectively. All assessments or experiments were carried out at 72 h after MCAO. B) T‐GSK significantly reduced the brain infarction evaluated by TTC staining at day 3 after the mouse MCAO. C) Quantitative analysis of cerebral infarction percentage calculated from TTC. (D and E) After mouse MCAO, the neurological recovery of mice in each group at different time points was evaluated by D) Longa score and E) rotarod test. F) Monitoring the effects of Sham, MCAO, GSK, NT‐GSK, and T‐GSK on the body weight of mice at different time points. (*n* = 3–5 per group). Data are shown as the mean ± SD.; two‐sided Student's t‐test was performed. ^****^ *p* < 0.0001 versus MCAO. ^####^ *p* < 0.0001 versus T‐GSK.

Inspired by the neuroprotective effects of T‐GSK, we set on to evaluate this protection in other models of brain injury. A mouse model of middle cerebral artery occlusion (MCAO) was created by inserting a filament with a silica gel tip into the middle cerebral artery. After 90 min of reperfusion, the filament was removed to induce reperfusion injury. The respective drugs were injected into mice that had been arbitrarily divided into treatment groups. **Figure** **5**A depicts a diagram of this model and the planned experiments. TTC staining of brain tissue sections revealed that MCAO caused a substantial amount of damage in comparison to the control group. The treatment with GSK, NT‐GSK, and T‐GSK mitigated this tissue damage (Figure 5B). Using a quantitative analysis of infarct volume, Figure 5C demonstrates that T‐GSK has the greatest potential to prevent ischemia/reperfusion injury compared to the other treatment groups. The Longa score was then utilized to evaluate the efficacy of our treatments against MCAO‐induced motor dysfunction and improved recovery (Figure 5D). The findings support the effectiveness of T‐GSK.

Subsequently, we conducted an experiment involving rotating rods to assess the neurological dysfunction of mice from different groups. Following MCAO surgery, the ability of mice to remain on a rotating rod was evaluated. As with the other treatment groups, the ability of mice in the MCAO group was severely impaired on day 1. The mice in treatment groups gradually regained this ability, with the T‐GSK group exhibiting the most significant recovery (Figure 5E). Throughout the duration of the experiment, the mice's body weights were monitored. Following MCAO, mice treated with GSK, NT‐GSK, or T‐GSK gradually regained their weight (Figure 5F).

### 2.10 Exploration of the Mechanistic Targets for T‐GSK

**Figure 6:**
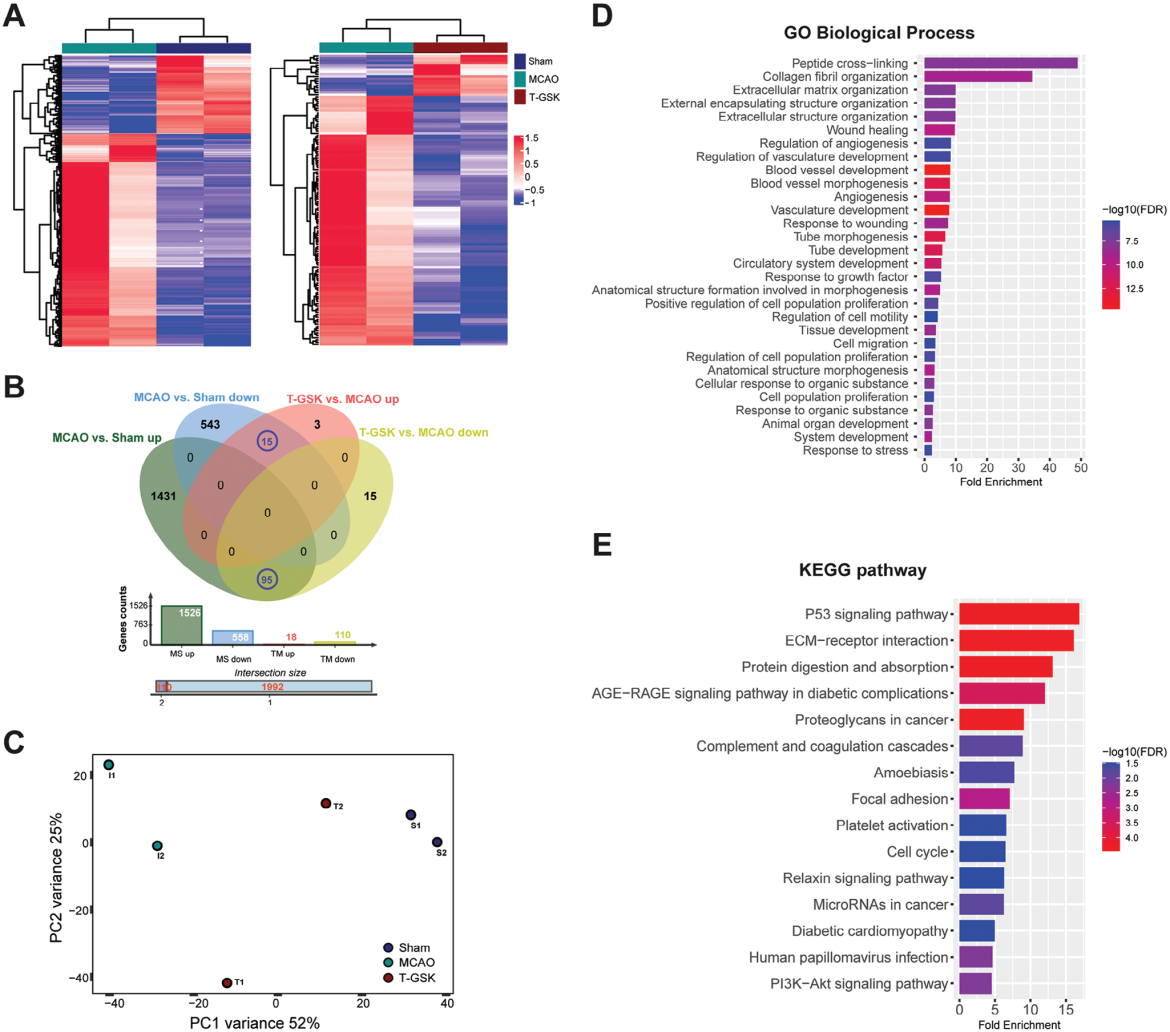
Identification of differentially expressed genes (DEGs). A) Heatmap showing differentially expressed genes in Sham, MCAO and T‐GSK. Two biological replicates are shown. B) Venn diagrams exhibit the overlaps of DEGs between MCAO versus Sham group and T‐GSK versus MCAO group. C) PCA analysis of Sham, MCAO and T‐GSK samples. D,E) The GO biological process (D) and KEGG pathway (E) enrichment of 110 DEGs (DU and UD genes).

To provide multiscale insights into T‐GSK treated ischemia‐reperfusion injury, we performed RNA‐seq of the injured brain tissues in Sham, MCAO, and T‐GSK treated mice with two biological replicates. Differentially expressed genes (DEGs) were determined by fold change >2.0 and adjusted P‐value < 0.05 (**Figure** **6**A). MCAO‐induced brain injury caused 2084 DEGs compared to Sham, including 1526 up‐regulated and 558 down‐regulated, whereas, T‐GSK treatment caused 128 DEGs compared to MCAO, including 110 down‐regulated and 18 up‐regulated (Figure 6B). Multivariate analysis (Principal Component Analysis, PCA) showed a closer distance between Sham and T‐GSK‐treated groups compared to MCAO (Figure 6C), indicating the efficacy of T‐GSK in reversing the changes in gene expression after injury. To characterize the key functional genes in the injury and treatment, we did overlaps among the DEGs, and interestingly identified 95 genes which were up‐regulated after MCAO and but down‐regulated after drug treatment (referred as UD pattern) as well as 15 genes which were down‐regulated after MCAO but up‐regulated after drug treatment (referred as DU pattern) (Figure 6B). The restored expression of these genes indicates that they may be the primary targets of GSK in protecting against cerebral ischemia‐reperfusion injury. The outstanding pathway analyzed by GO (Figure 6D) and KEGG (Figure 6E) pathway analysis was related to extracellular matrix remodeling which was critical for NET‐associated inflammation,^[^ ^31^, ^32^ ^]^ indicating that nanoparticles may alleviated the brain injury through remodeling extracellular matrix.

## 3 Conclusion

Our research demonstrates that this nanocarrier drug delivery system has significant potential for treating TBI and stroke. Under normal conditions, neutrophils cannot cross the BBB. The BBB is however compromised by traumatic brain injury and stroke, allowing neutrophils to enter the brain. In experimental models of TBI and MCAO, intravenous administration of targeted nanomedicine (T‐GSK) diminished NETosis and alleviated these symptoms. Flow cytometry and NIR‐II imaging confirmed the uptake and brain‐specific delivery of nanomedicine by neutrophils. T‐GSK exhibited notable anti‐oxidative, anti‐apoptotic, and anti‐inflammatory properties. By reducing ROS levels and activating astrocytes and microglia, T‐GSK successfully reduced neuroinflammation following brain injury. Our findings may serve as a basis for the design, development, and application of novel nano‐drug delivery systems in future clinical and experimental studies. This research elucidates the cellular and molecular mechanisms by which targeted nanomedicines can achieve drug enrichment at the site of injury, limit brain damage, and improve cerebrovascular function.

## 4 Experimental Section

### The Correlation Study between Neutrophils and Neurological Function in Patients with Severe Brain Injuries

The blood samples were performed following a protocol approved by Medical Ethics Committee of The Gaozhou People's Hospital (approval number: GYLLPJ‐2023020). The neutrophil counts were tested by collecting blood samples from patients with TBI (*n* = 13) and stroke (*n* = 10).

### Preparation of T‐GSK and NT‐GSK NPs

For T‐GSK NPs, the DSPE‐Se‐Se‐PEG_2K_‐NHS (80 mg) and formyl peptide receptor (FPR) targeting peptide cinnamyl‐F‐(D) L‐F‐(D) L‐F (CFLFLF) (30 mg) were dissolved in CH_2_Cl_2_ and added with DIPEA (2 mg), which were incubated for 24 h. The product DSPE‐Se‐Se‐Pep was then purified by HPLC. A solution of GSK (4 mg) and DSPE‐Se‐Se‐Pep (40 mg) in THF (2 mL) was added into ultrapure water (2 mL) dropwise under stirring and kept in a fume hood overnight to evaporate the THF. For T‐GSK‐ICG, a solution of DSPE‐Se‐Se‐Pep (40 mg) and GSK (4 mg) in THF (2 mL) was added into ICG aqueous solution (2 mL, 1 mg mL^−1^) dropwise under stirring and kept in a fume hood overnight to evaporate the THF. The obtained T‐GSK and T‐GSK‐ICG nanoparticles were purified via dialysis (MWCO, 3000 Da) and stored at 4 °C for the following use. The non‐targeting nanoparticles NT‐GSK NPs were prepared by the similar methods.

### Animal Model

The protocol used for the animal experiments was authorized by the Animal Care and Use Committee of Zhejiang University (approval number: ZJU20230221). The Animal Center of Zhejiang University (Zhejiang, China) provided adult male C57BL/6 mice weighing 20–25 g and aged 6–8 weeks. They were kept under a 12 h light/dark cycle access to water and food ad libitum. The details about the development of TBI and MCAO mouse models are given in the Supporting Information.

### NIR‐II Imaging

All NIR‐II images were collected on MARS in vivo imaging system (Artemis Intelligent Imaging, Shanghai, China). Sham group mice were injected with T‐GSK‐ICG via the tail vein, and the TBI mice were similarly injected with NT‐GSK‐ICG and T‐GSK‐ICG. 1, 4, 8, and 24 h after administration, the brain tissue was exposed to the NIR‐II imaging system (808 nm, 50 mWcm^−2^) (*n* = 3/group). Time‐dependent NIR‐II fluorescence images of brain tissue were gained by using in vivo imaging system. The excitation was induced by an 808‐nm laser and was filtered with an 808 nm long‐pass filter. An 1100‐nm bandpass filter was used to collect fluorescence emission and the exposure time was 50 ms (*n* = 3/group).

### Longa Test and Rotarod Test

The Longa test was used to assess the neurobehavioral impairment caused by an ischemic stroke (*n* = 3–5/group). On a scale of 0–14, each score represented the extent of neurological impairment: 0: normal, no neurological deficit; 1: inability to fully extend the left forelimb, mild neurological deficit; 2: unable to walk in a straight line, moderate neurological deficit; 3: Turn to the right side (hemiplegia side), severe neurological deficit; 4 points: spontaneous loss of ability to walk, loss of consciousness. To evaluate balance and motor coordination rotarod test was performed (*n* = 6/group). The mice received a 2‐day training prior to TBI/MCAO, which steadily increased from 4 to 20 rpm in 5 min. The mean time of latency to fall was measured when the mice fall off the rod in three trails.

### TTC Staining

The mice were sacrificed under deep anesthesia on day 3 (*n* = 3/group). Brains were sliced into six 2 mm thick sections and incubated in 2% 2,3,5‐triphenyltetrazolium chloride (TTC) (LEAGENE, Beijing, China) solution at 37 °C for 20 min. The sections were then photographed, and the Infarct lesion area was calculated using Image J analysis software. Infarcts were recognized as the unstained areas of brain sections. Infarct size was indirectly determined by subtracting the stained area (non‐infarcted) area in the infarcted hemisphere from the total area in the non‐infarcted hemisphere. The percentage of hemispheric infarct area was calculated according following equation: infarct volume/total volume of non‐infarcted hemisphere × 100%.

### MRI Imaging

On the third day following reperfusion, TBI mice in different treatment groups were anaesthetized with isoflurane (*n* = 3/group) and were subjected to T2‐weighted coronal magnetic resonance imaging (MRI) of the brain with 3.0 T MR scanner (Siemens, Trio, Germany) with a 7.0 T CG NOVILA system (Chenguang Med Tech Co, Shanghai, China). Finally, the infarct volume from high‐signal areas were analyzed by Image J.

### EB Permeability Assay

The BBB disruption was assessed by the EB (MACKLIN, Shanghai, China) exudation technique. On day 3 post TBI/MCAO, intravenous injection (via tail vain) of 4% EB (2.5 ml/kg) was performed. After 6 h, mice in different groups were then transcardially perfused with heparinized saline (*n* = 3/group). Brains were then photographed before dividing them into two hemispheres. Each hemisphere was homogenized in 1 mL of 50% trichloroacetic acid and centrifuged (Ke Cheng, Suzhou, China) for 20 min at 14 000 rpm at 4 °C. The supernatant was collected and its absorbance at 610 nm was measured using spectrophotometry. The EB content from each brain was then calculated.

### Brain Water Content

The brain water content was calculated using the wet‐dry method. On the 3rd day after TBI, mice were sacrificed, and the brain tissues were taken (*n* = 3/group). The wet weight of the brain tissue was immediately weighed and dried at 100 °C for 2 days to determine the dry weight. Brain water content was calculated as follows: (wet weight‐dry weight)/wet weight × 100%.

### Immunofluorescence Staining, ROS detection, TUNEL Staining, and HE Staining

Three days post TBI, the mice were anesthetized and perfused with PBS, followed by 4% paraformaldehyde perfusion (*n* = 3/group). The brains were then removed and kept overnight in 4% paraformaldehyde. The brain tissues were subsequently embedded in paraffin wax and sliced into 5‐µm thick sections using a microtome (Leica, Heidelberg, Germany). The coronal sections were stained with primary antibodies: Rabbit Anti‐Iba‐1, GFAP, and Ly6G (Servicebio, Wuhan, China) overnight at 4 °C. Next, the coronal sections were stained with a secondary antibody (Servicebio, Wuhan, China) and the nuclei were counterstained DAPI (Servicebio, Wuhan, China). These brain tissues were visualized under fluorescent microscope and images were captured. IF staining results were quantified via image J. by calculating the integrated density that represents the sum of the pixel values in the immunofluorescent microscopic image. The integrated density of sham group was defined as 1 and then the relative integrated density for other groups was then calculated using the following formula: Integrated density of each group / integrated density of the sham group (i.e., 1). The results were plotted into histogram using GraphPad Prism 8.

Next, to determine the ROS level, the coronal sections were stained with ROS staining solution (Servicebio, Wuhan, China) and incubated at 37 °C for 30 min in dark. To detect the apoptotic cells, TUNEL reactivity was measured using TUNEL Apoptosis Assay Kit (Servicebio, Wuhan, China). Then tissue sections were then incubated with DAPI solution at room temperature for 10 min in dark and visualized under a fluorescence microscope (Leica, Heidelberg, Germany). The vital organs, including heart, lungs, liver, kidney, and spleen were subjected to freeze‐cut into 5 µm thick coronal sections and stained with Hematoxylene & Eosin (HE).

### Enzyme‐Linked Immunosorbent Assay (ELISA) for Inflammatory Cytokine Determination

Three days after successful TBI modeling, brain tissues of deeply anaesthetized mice in different groups were obtained and homogenized (*n* = 3/group). Homogenized tissues were subjected to centrifugation for 15 min at 500 × g, and supernatants were collected. Different reagents were added as per manufacturer's instruction (Fine test, Wuhan, China) to measure TNF‐α and IL‐6 in the supernatants (*n* = 3/group) using a microplate reader. The results were analyzed by drawing a standard curve and calculating the concentrations of TNF‐α and IL‐6 accordingly.

### Cellular Uptake

T‐GSK‐ICG and NT‐GSK‐ICG uptake by neutrophils was investigated by flow cytometry and confocal laser scanning microscopy (CLSM). Briefly, freshly isolated neutrophils were cultured on lysine‐coated coverslips in 12‐well plates at the density of 1 × 105 cells per well and incubated for 12 h (*n* = 3/group). Then, 2 µg mL^−1^ of ICG, T‐GSK (T‐GSK‐ICG) and NT‐GSK (NT‐GSK‐ICG) were added to respective groups and incubated for 24 h. Finally, nuclei were stained with DAPI and the binding of nanoparticles was viewed under CLSM. The binding of nanoparticles to neutrophils was quantified by flow cytometry. To this end, neutrophils were cultured in 12‐well plate (3 × 106 cells/well) and treated as described above. Following indicated treatments, the cells were collected, stained with DAPI, and run through flow cytometry to quantify NPs uptake (FACSCalibur, Becton Dickinson, USA).

Similarly, human neutrophils were isolated from blood collected from the healthy individuals. Isolated neutrophils were seeded on lysine‐coated coverslips placed in 12‐well culture plates and treated with ICG‐labeled T‐GSK (T‐GSK‐ICG). The binding of T‐GSK‐ICG with neutrophils was then visualized using CLSM. Quantification of nanoparticles binding with human neutrophils was done by flow cytometry.

### Measurement of Intracellular ROS

PC12 cells at logarithmic growth stage were seeded into 24 well plates, at a seeding density of 4 × 104 cells per well. The cultured cells were randomly divided into five groups: control group, H_2_O_2_ group, H_2_O_2_+GSK group, H_2_O_2_+NT‐GSK group, and the H_2_O_2_+T‐GSK group (*n* = 3/group). After being exposed to 100 mm H_2_O_2_ for 30 min, the drugs (150 µm L^−1^) were added to the culture plate for 24 h. According to the manufacturer's instructions (GLPBIO, Nantong, China), diluted DCFH‐DA (10 µm) was added to each well and incubate at 37 °C and 5% CO_2_ for 30 min in the dark. Afterward the cells were washed twice with PBS, the fluorescence intensity (indicating ROS generation) of the cells in each group was observed under a fluorescence microscope, photographed, and analyzed.

### RNA‐Seq Library Construction and Data Analysis

C57BL / six mice aged 10–12 weeks were randomly divided into Sham, MCAO, and T‐GSK groups. The middle cerebral artery occlusion (MCAO) was performed to establish the mouse model of cerebral ischemia‐reperfusion injury in both MCAO and T‐GSK groups, while in Sham operated mice only the epidermis was incised. Brain tissues from mice in each group (*n* = 2) were processed for RNA extraction using RNA extraction kit according to manufacturer's instructions. The extracted RNA samples were sent to Shanghai meisequence Biotechnology Co., Ltd for transcriptomic sequencing. Differential analysis between groups was carried out using the deseq2 package. The differentially expressed genes between Sham versus MCAO group and MCAO versus T‐GSK group were screened by using |log2 FC| > 1, adjusted P‐value < 0.05. PCA analysis was performed on count data samples, and the results were displayed by ggplot2. Expression levels of genes were normalized by FPKM (Fragments Per Kilobase of transcript per Million) method, and a dynamically expressed gene in three sequential stages was computed by the Z‐score of its relative abundance in three stages, where the summit of the expression abundance was defined as the Z‐score >0.5 at the stage while <−0.5 at the others, and the nadir of expression abundance was vice versa. dynamic expression patterns were categorized into four classes: DD, DU, UD, and UU, in which D stands for down‐regulation and U stands for up‐regulation. GO and KEGG enrichment analysis was carried out using ShinyGO.

### Statistical Analyses

The independent two‐sample t‐tests was used to compare two groups, while one‐way analysis of variance (ANOVA) was used for comparison between multiple groups: sham, PBS, GSK, NT‐GSK, and T‐GSK groups. If the ANOVA test produced significant results, Tukey's test was applied to make pairwise comparisons between groups. All the statistical analysis was performed using GraphPad Prism 8 (GraphPad Software, USA), and the graphs were plotted. The results are presented as the mean ± SD, and a P‐value < 0.05 was considered to be statistically significant.

## Conflict of Interest

The authors declare no conflict of interest.

## Supporting information

Supporting Information

## Data Availability

The data that support the findings of this study are available in the supplementary material of this article.
