# Supplementary material for: Neutrophil Targeting Platform Reduces Neutrophil Extracellular Traps for Improved Traumatic Brain Injury and Stroke Theranostics
Source: Adv Sci (Weinh). 2024 Mar 23;11(21):2308719. doi: 10.1002/advs.202308719 (PMC11151022; doi:10.1002/advs.202308719)
Supplement: Supplementary file 1 — Supporting Information [file ADVS-11-2308719-s001.pdf]

ADVANCED SCIENCE Open Access

Supporting Information

for *Adv. Sci.*, DOI 10.1002/advs.202308719

# Neutrophil Targeting Platform Reduces Neutrophil Extracellular Traps for Improved Traumatic Brain Injury and Stroke Theranostics

*Qingchun Mu*, Kai Yao, Madiha Zahra Syeda, Jinlong Wan, Qian Cheng, Zhen You, Rui Sun, Yufei Zhang, Huamiao Zhang, Yuting Lu, Zhicheng Luo, Yang Li, Fuyao Liu, Huiping Liu, Xinyu Zou, Yanfen Zhu, Kesong Peng, Chunming Huang, Xiaoyuan Chen* and Longguang Tang**

# Supporting Information

**Neutrophil targeting platform reduces neutrophil extracellular traps for improved traumatic brain injury and stroke theranostics**

*Qingchun Mu[#,]*, Kai Yao[#], Madiha Zahra Syeda[#], Jinlong Wan, Qian Cheng, Zhen You, Rui Sun, Yufei Zhang, Huamiao Zhang, Yuting Lu, Zhicheng Luo, Yang Li, Fuyao Liu, Huiping Liu, Xinyu Zou, Yanfen Zhu, KeSong Peng, Chunming Huang, Xiaoyuan Chen*, Longguang Tang**

Dr. Q. Mu, Dr. MZ. Syeda, J. Wan, Z. Luo, X. Zou, Prof. C. Huang

Gaozhou People's Hospital, Maoming 525200, China

Email: muq@hainmc.edu.cn

Dr. K. Yao

Department of Neurosurgery, First Affiliated Hospital of Harbin Medical University, Harbin 150001, China

Dr. MZ. Syeda

St. Michael's Hospital, fully affiliated hospital of University of Toronto, Toronto, Ontario, M5B 1W8, Canada

H. Zhang, Y. Lu, Y. Li, H. Liu, Prof. Y. Zhu, Prof. K. Peng, Prof. L. Tang
Department of Pharmacy, Center for Regeneration and Aging Medicine, the Fourth Affiliated Hospital of School of Medicine, and International School of Medicine, International Institutes of Medicine, Zhejiang University, Zhejiang-Denmark Joint Laboratory of Regeneration and Aging Medicine, Yiwu, 322000, China

Email: tanglongguang@zju.edu.cn

Q. Cheng, Prof. Y. Zhang,

Basic Medical College, Guilin Medical University, Guilin 541199, China

Z. You

Liangzhu Laboratory, Zhejiang University, 1369 West Wenyi Road, Hangzhou 311121, China

R. Sun

School of Pharmaceutical Sciences, Guangdong Provincial Key Laboratory of New Drug Screening, Southern Medical University, Guangzhou 510515, China

Prof. Fuyao Liu,

Key Laboratory for Advanced Drug Delivery Systems of Zhejiang Province, College of Pharmaceutical Sciences, Zhejiang University, 310058, Hangzhou, China

Prof. Xiaoyuan Chen,

Departments of Diagnostic Radiology, Chemical and Biomolecular Engineering, and Biomedical Engineering, Yong Loo Lin School of Medicine and College of Design and Engineering, National University of Singapore, Singapore, 119074, Singapore.

Clinical Imaging Research Centre, Centre for Translational Medicine, Yong Loo Lin School of Medicine, National University of Singapore, Singapore, 117599, Singapore.

Nanomedicine Translational Research Program, Yong Loo Lin School of Medicine, National University of Singapore, Singapore, 117597, Singapore.

Email: chen.shawn@nus.edu.sg

[#] These authors contributed equally to this work.

**Supplementary figures**

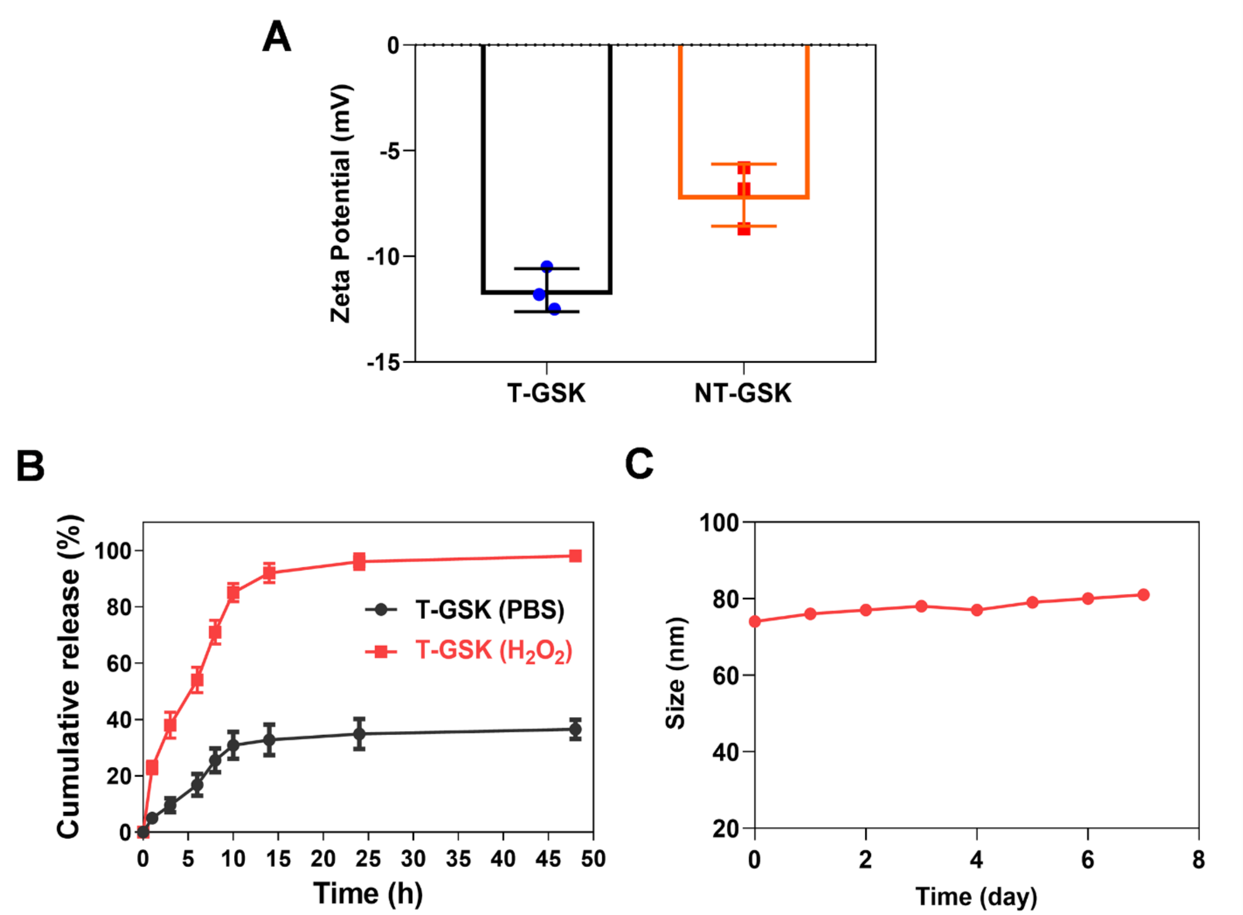


**Figure S1.** Materials Characterization. (A) The zeta potential of NT-GSK and T-GSK nanoparticles. (B) Cumulative release of GSK from NPs in PBS (7.4 pH) and $H_2O_2$ (1 mM). (C) Stability of T-GSK nanoparticles (NPs) after a week storage in aqueous solution under 4 ℃.

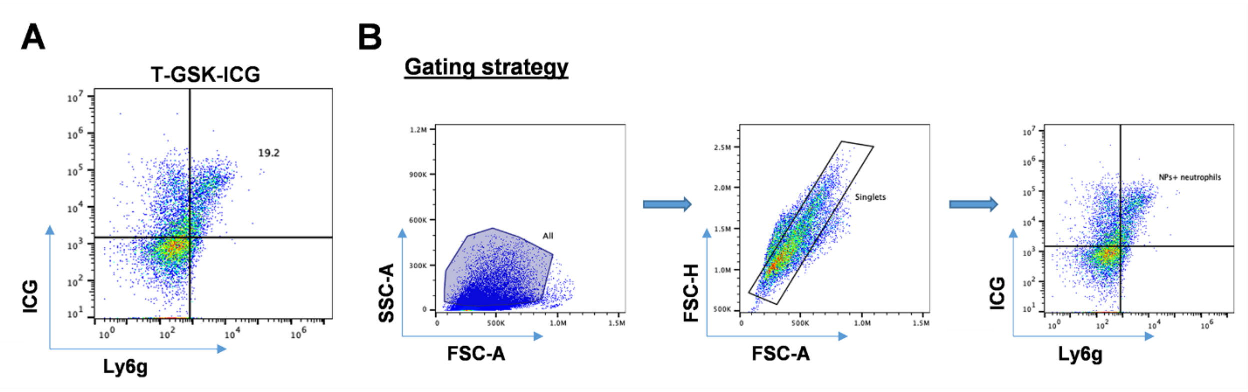


**Figure S2**. Activated neutrophils uptake ICG-labeled T-GSK NPs. (A) Flow cytometry analysis of the ICG-labeled nanoparticles uptake by neutrophils. (B) Gating strategy: Forward (FSC-A) and side scatter (SSC-A) gating was used to identify the single cells (singlets). To identify the population of interest (neutrophils bound nanoparticles), singlets stained with Ly6G (neutrophils surface marker) and ICG (ICG dye-labeled nanoparticles), were identified as a double positive population (Ly6G+ICG+).

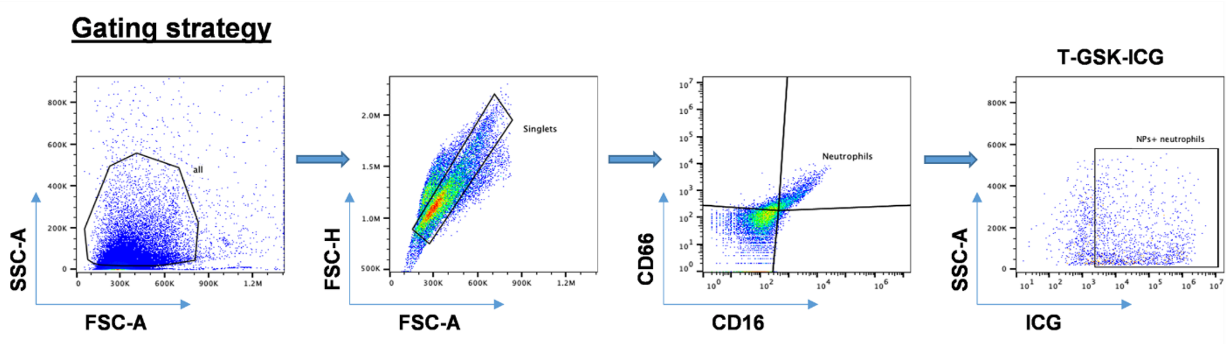


**Figure S3.** Binding of ICG-labelled nanoparticles on human neutrophils. Flow cytometry analysis of the ICG-labeled NPs (T-GSK-ICG) uptake by human neutrophils and the gating strategy.

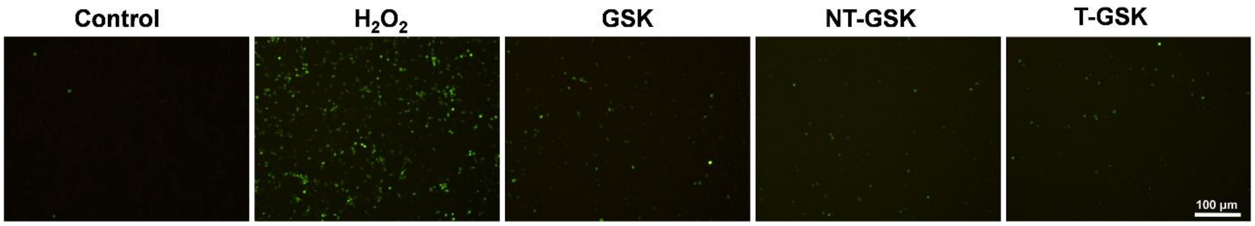


**Figure S4.** The expression of ROS inPC12 cells of different groups was detected by DCFH-DA experiment. Immunofluorescence results showed that the targeting drug significantly reduced ROS production in PC12 cells induced by hydrogen peroxide ($H_2O_2$)

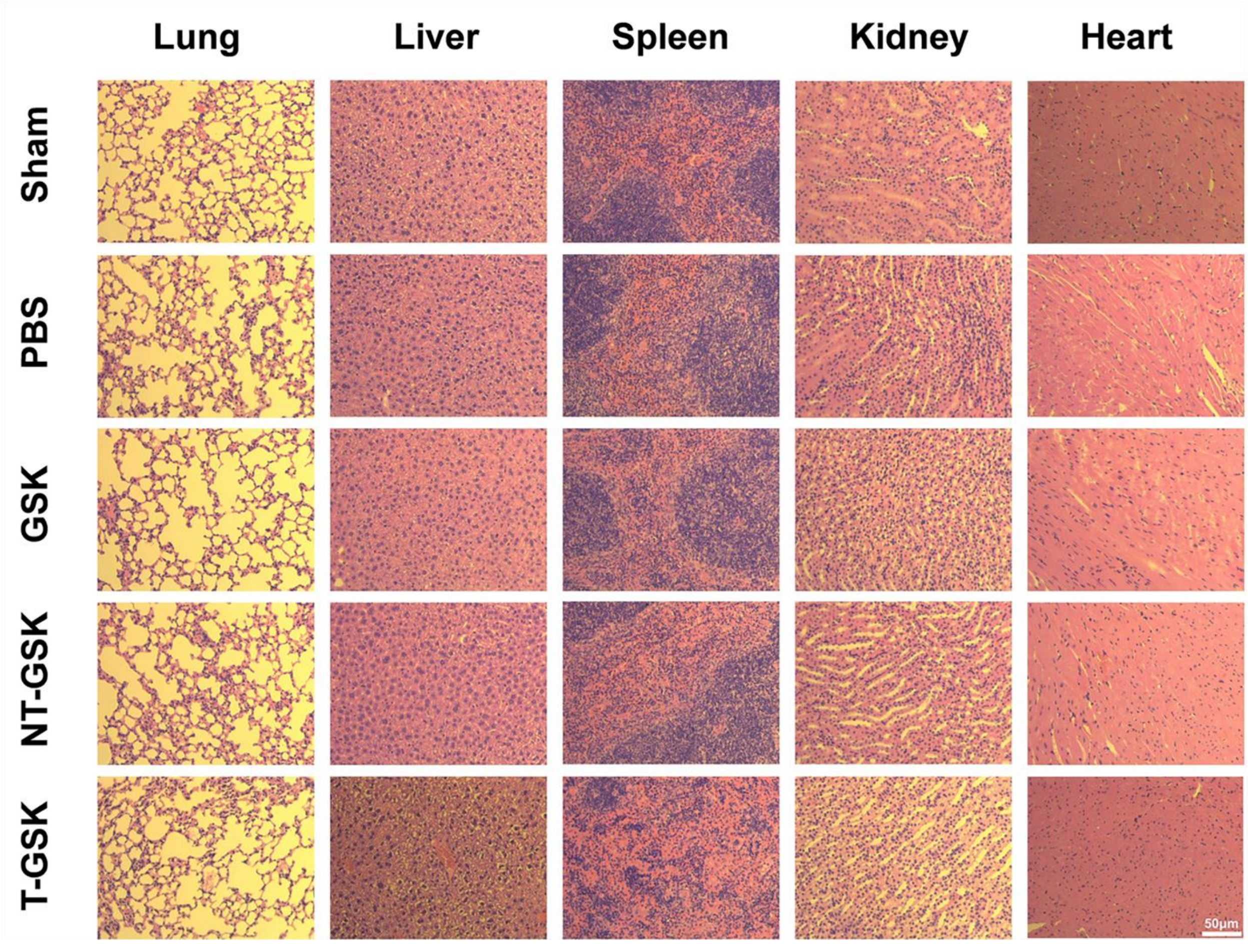


**Figure S5.** HE Staining of lung, liver, spleen, kidney, and heart showed no histology differences in Sham, PBS, GSK, NT-GSK, and T-GSK groups.

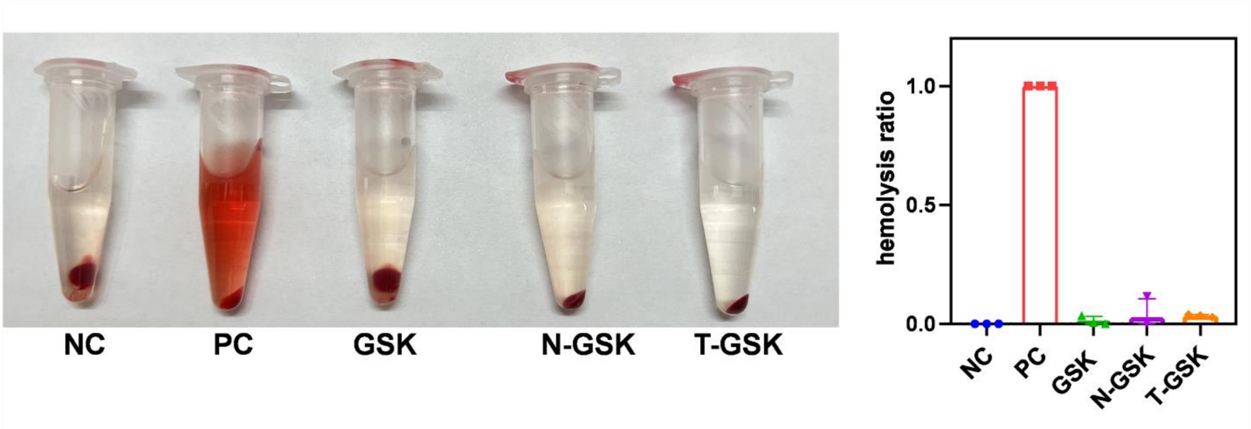


**Figure S6.** Blood sample was treated with GSK, NT-GSK and T-GSK for 3 hours at room temperature to measure blood hemolysis. PBS was used as negative control (NC), and dd$H_2O$ was used as positive control (PC).

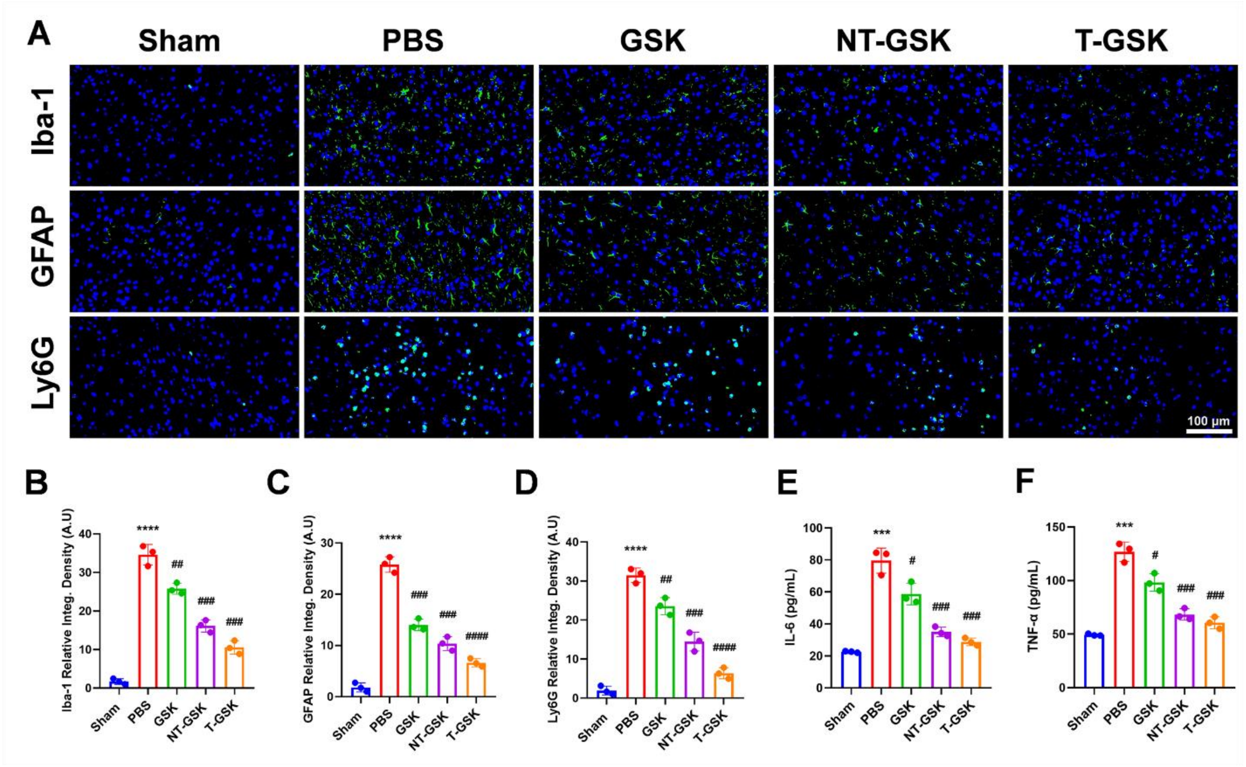


**Figure S7. Anti-inflammatory effects of T-GSK on the progression TBI induced neuroinflammation.** (A) Immunofluorescence assays confirmed the activation of microglia, astrocytes, and neutrophils after TBI in different treatment groups. (B-D) Quantitative analysis showed that T-GSK could significantly reduce Microglia (B), Astrocytes (C), and neutrophils (D), and inhibited the production of IL-6 (E), and TNF-α (F). ***$P < 0.001$ versus Sham, ****$P < 0.0001$ versus Sham, ##$P < 0.01$ versus PBS, ###$P < 0.001$ versus PBS, ####$P < 0.0001$ versus PBS.

## Supplementary methods

**TBI Model:** Mice were randomly assigned to the following experimental groups before surgery: Sham, TBI, GSK, NT-GSK, and T-GSK. First, GSK was dissolved in DMSO and then diluted in PBS to a final concentration of 1 mg/mL. The mice were anesthetized with 2% isoflurane. After depilation and disinfection, they were placed in a stereotaxic apparatus. Lidocaine (0.05 mL, 5 mg mL−1) and bupivacaine (0.05 mL, 0.3 mg $mL^{-1}$) were injected into their scalp. Based on the model described by Feeney *et al*[1]., with certain modifications, a midline incision was made over the scalp, and a 5 mm × 5 mm craniotomy was performed on the left parietal skull (3 mm right of the sagittal

suture and 3 mm behind the coronal suture). A beating device was fixed on the stereotactic frame, and a weight of 20 g with a diameter of 5 mm and a length of 5.5 cm was dropped from a distance of 15 cm to produce TBI[1]. A gelatin sponge was used, and the scalp incision was sutured until there was no more active bleeding. The Sham operation group underwent anesthesia and craniotomy without brain damage. When the anesthetized mice gradually recovered under a heat lamp, they were returned to their cages. Mice were weighed on days 0, 1, 3, 7, and 14 after TBI.

**MCAO Model:** Briefly, mice were anesthetized with 2% isoflurane. After making a midline incision in the neck, the right common carotid, external and internal carotid arteries were dissected. A 6-0 silica-coated nylon monofilament was inserted into the external carotid artery and advanced along the internal carotid artery and occluded the origin of the middle cerebral artery. After 90 minutes of occlusion, the monofilament was removed to allow for reperfusion. The incision was sutured, and mice were allowed to recover when they were kept warm under a heating lamp. Mice were weighed at different time points after MCAO. Mice were randomly assigned to the following experimental groups before surgery: Sham, MCAO, GSK, NT-GSK, and T-GSK. First, GSK was dissolved in DMSO and then diluted in PBS to a final concentration of 1 mg/mL. Free GSK or GSK nanoparticles (T-GSK, NT-GSK) (8 mg/kg of GSK) were administered via the tail vein immediately, on day 1, and 2 after MCAO. The sham group was established, consistent with the treatment of MCAO group except for the occlusion. The MCAO group injected an equal volume of PBS compared with other experimental groups.

**Flow cytometry of NETs:** Mouse blood collected from different groups was diluted in PBS and stained with CD45-FITC (BioLegend, USA), CD11B-PE (BioLegend, USA) and Ly6G-Per-CP (Tonbo Biociences, USA) after TBI on day 3. After the samples were centrifuged at 300 × g for 5 min, cells were fixed in paraformaldehyde for 45 min and permeabilized by TrixionX-100 for 10 min. After the samples were centrifuged at 300 × g for 5 min, cells were then permeabilized by TrixionX-100 and stained with Anti-MPO/APC (Bioss, Beijing, China) and Anti-Histone H3 (CitH3)/PE (Bioss, Beijing, China). At last, the stained cells were analysed by flow cytometry (FACSCalibur, Becton Dickinson, USA) following the manufactrurer's instrraction.

**mNSS:** Neurological function was calculated using the motified neurological severity scale (mNSS). Mice were subjected to motor function (muscle state and abnormal activities), sensation (visual, tactile, and balance), and reflection after TBI. The scores were recorded when the task was not completed or the corresponding reflex was lost. The mNSS is scored on a scale of 0-18, with a total score of 18 indicating severe neurological impairment, a score of 0 indicating normal performance, a score of 13-18 indicating severe injury, a score of 7-12 indicating moderate injury, and a score of 1-6 indicating mild injury. Neurological deficits were measured by blinded investigators at different time points.

**Immunofluorescence Staining of NETs:** The mice were sacrificed on day-3 after TBI, the brain tissues were taken out and embedded in paraffin wax. 5-μm thick slices were cut by microtome (Leica, Heidelberg, Germany). After dewaxed, the slices were placed in BSA solution for 30 minutes. Then the slices were incubated with primary antibodies: diluted Rabbit Anti Ly6G (Servicebio, Wuhan, China), and Histone H3 Antibody (CitH3) (Abways, Shanghai, China) overnight at 4 °C. Then the secondary antibody (Servicebio, Wuhan, China) were added and incubated for 50 min. Finally, the slices were placed in PBS and washed three times and stained with DAPI at room temperature in dark for 10 minutes.

1. Feeney, D. M.; Boyeson, M. G.; Linn, R. T.; Murray, H. M.; Dail, W. G., Responses to cortical injury: I. Methodology and local effects of contusions in the rat. *Brain Res* **1981,** *211* (1), 67-77.
